# Immunoreactive trypsinogen levels in newborn screened infants with an inconclusive diagnosis of cystic fibrosis

**DOI:** 10.1186/s12887-019-1756-4

**Published:** 2019-10-22

**Authors:** Chee Y. Ooi, Rosie Sutherland, Carlo Castellani, Katherine Keenan, Margaret Boland, Joe Reisman, Candice Bjornson, Mark A. Chilvers, Richard van Wylick, Steven Kent, April Price, Dimas Mateos-Corral, Daniel Hughes, Melinda Solomon, Peter Zuberbuhler, Janna Brusky, Peter R. Durie, Felix Ratjen, Tanja Gonska

**Affiliations:** 1 Discipline of Paediatrics, School of Women’s and Children’s Health, Faculty of Medicine, University of New South Wales, Sydney, Australia; 2 miCF Research Centre and Department of Gastroenterology, Sydney Children’s Hospital Randwick, High Street, Randwick NSW, Sydney, Australia; 3 Department of Paediatrics, Division of Gastroenterology, Hepatology and Nutrition, The Hospital for Sick Children, Toronto, Ontario Canada; 4 Cystic Fibrosis Centre, Azienda Ospedaliera Universitaria Integrata di Verona, Verona, Italy; 5 Physiology and Experimental Medicine, Research Institute, The Hospital for Sick Children, Toronto, Ontario Canada; 6 Division of Gastroenterology, Hepatology and Nutrition, Children’s Hospital of Eastern Ontario, Ottawa, Canada; 7 Department of Paediatrics, Division of Respirology, Children’s Hospital of Eastern Ontario, Ottawa, Canada; 8 Department of Pediatrics, Section of Respiratory Medicine, University of Calgary, Alberta Children’s Hospital, Calgary, Alberta Canada; 9 Department of Pediatrics, Division of Pediatric Respiratory Medicine, BC Childrens Hospital, Vancouver, BC Canada; 10 Department of Pediatrics, School of Medicine, Queen’s University, Kingston, Ontario Canada; 11 Victoria General Hospital, Victoria, BC Canada; 12 Children’s Hospital Of Western Ontario, London, Ontario Canada; 13 IWK Health Centre, Halifax, Nova Scotia Canada; 14 Department of Paediatrics, Division of Respiratory Medicine, The Hospital for Sick Children, Toronto, Canada; 15 Stollery Children’s Hospital, Edmonton, Alberta Canada; 16 Royal University Hospital, Saskatoon, Saskatchewan Canada

**Keywords:** CF screen positive inconclusive diagnosis (CFSPID), CFTR-related metabolic syndrome (CRMS), Newborn screening, Sweat test, Trypsinogen

## Abstract

**Background:**

Newborn screening (NBS) for cystic fibrosis (CF) not only identifies infants with a diagnosis of CF, but also those with an uncertain diagnosis of cystic fibrosis (CF), i.e. CF transmembrane conductance regulator (CFTR)-related metabolic syndrome (CRMS) or CF screen positive inconclusive diagnosis (CFSPID). These infants have an uncertain long-term outcome and it is currently unclear around time of diagnosis, which infants are at higher risk of later fulfilling a CF diagnosis. In this study, we hypothesised that immunoreactive trypsinogen (IRT) levels, used in NBS as a marker of pancreatic disease and function, may reflect the degree of CFTR dysfunction in each individual and therefore would help to identify those with CRMS/CSPID who are later at risk for meeting the criteria of CF.

**Methods:**

In this longitudinal, prospective study, infants with CRMS/CFSPID and CF were recruited and followed in 9 CF clinics (Canada and Italy). We compared NBS IRT levels between CF and CRMS/CFSPID, and between children with CRMS/CFSPID→CF and CRMS/CFSPID→CRMS/CFSPID during the period of June 2007 to April 2016.

**Results:**

Ninety eight CRMS/CFSPID and 120 CF subjects were enrolled. During the study period, 14 (14.3%) CRMS/CFSPID subjects fulfilled the diagnostic criteria for CF (CRMS/CFSPID→CF), while the diagnosis remained uncertain (CRMS/CFSPID→ CRMS/CFSPID) in 84 (85.7%) subjects. Significantly higher NBS IRT concentrations (ng/ml) were present in CF than CRMS/CFPSID (median (interquartile range): 143.8 (99.8–206.2) vs. 75.0 (61.0–105.9); *P* < 0.0001). Infants with CRMS/CFSPID→CF (*n* = 14) had significantly higher NBS IRT concentrations (ng/ml) than CRMS/CFSPID→ CRMS/CFSPID (*n* = 83) (median (interquartile range): 108.9 (72.3–126.8) vs. 73.7(60.0–96.0); *P* = 0.02).

**Conclusions:**

Amongst infants who tested positive on NBS for CF, there is a gradation of elevated NBS IRT concentrations. Infants with CF have higher NBS IRT levels than CRMS/CFPSID, and higher NBS IRT concentrations were present in infants with CRMS/CFSPID→CF than CRMS/CFSPID→ CRMS/CFSPID. NBS IRT concentrations, in concert with other factors, may have the potential to predict the likelihood of CF amongst infants with CRMS/CFSPID.

## Background

Cystic fibrosis (CF) is a life-limiting, autosomal recessive disease caused by pathogenic mutations in the gene encoding the CF transmembrane conductance regulator (CFTR) protein. With the availability and expansion of newborn screening (NBS) programs for CF, a new subgroup of infants with a positive NBS result, but an inconclusive diagnosis of CF has emerged. These infants, who have an uncertain long-term outcome (CF vs. CFTR-related disorder vs. healthy), have been designated as CF transmembrane conductance regulator (CFTR)-related metabolic syndrome (CRMS) or CF screen positive inconclusive diagnosis (CFSPID) (also known as CRMS/CFSPID in the most recent international joint consensus) [1, 2].

Since the first report of newborns with CF having elevated immunoreactive trypsinogen (IRT) levels [3], IRT measurements have become central to NBS protocols worldwide. Immunoreactive trypsinogen levels reflect severity of pancreatic disease and pancreatic function [4–6], which in turn is associated with the severity of the CFTR gene defect. Higher and more rapid decline in IRT levels are associated with more severe CFTR variants (Class I-III), while lower levels are observed with less severe variants (Class IV-VI) [7–9]. There are also non-CFTR related factors that can lead to a high neonatal IRT level, including stressful or prolonged labor, Black race, and trisomy 21 [1]. We hypothesised that IRT levels may also reflect the severity of the CFTR gene defect among children with CRMS/CFSPID and would be different between children with CRMS/CFSPID who subsequently met the criteria for CF (CRMS/CFSPID→CF) and those whose diagnosis remained uncertain (CRMS/CFSPID→ CRMS/CFSPID). In this study, we compared IRT measured at NBS to differentiate between CF and CRMS/CFSPID, and between children with CRMS/CFSPID who subsequently met the criteria for CF (CRMS/CFSPID→CF) and those whose diagnosis remained uncertain (CRMS/CFSPID→ CRMS/CFSPID).

## Materials and methods

### Subjects

A clinical-research protocol was developed to evaluate and follow infants with a diagnosis of CRMS/CFSPID in a prospective, longitudinal manner from 9 CF clinics across 5 provinces in Canada (Ontario, British Columbia, Alberta, Saskatchewan and Nova Scotia) and from Verona, Italy. Interim data for the period between June 2007 and April 2016 are reported here. Some of the subjects have been previously reported in a separate study [10].

As part of NBS protocol, dried blood spots were collected in the first 2 days of life. Analyses for IRT levels from these samples were performed using the AutoDELFIA method (Perkin-Elmer Life Sciences, Boston, USA). Accordingly, an infant was considered to be NBS-positive if: [1] IRT exceeded the site-specific cut-off, in addition to at least one *CFTR* mutation (and/or raised meconium lactase in Verona, Italy); or, [2] IRT exceeded the 99.9th centile when no mutations were identified. All infants who were identified as NBS-positive were then referred to their local CF centre for sweat testing and genotyping to determine whether they fulfilled the diagnostic criteria for CF (based on sweat chloride ≥60 mmol/L and/or CF-causing mutations on both alleles) [1].

**Fig. 1:**
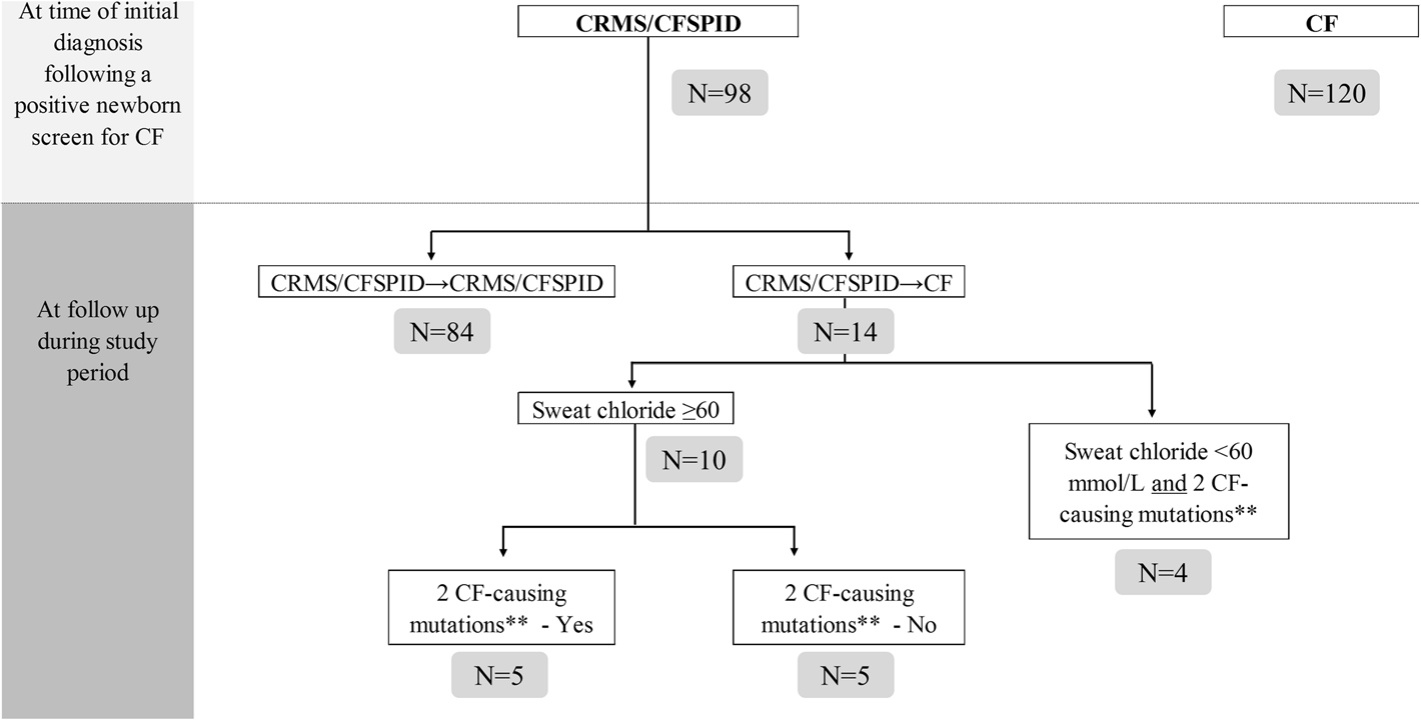
Flowchart of the study summarising time of initial diagnosis and during the study follow-up period

This study was designed intentionally to reflect real-life experiences in the CF clinic following a positive NBS, during which parents/carers are informed as to whether their infant has a diagnosis of CF or CRMS/CFSPID (Fig. 1). For the latter, NBS-positive infants at each centre were defined and entered into the study as CRMS/CFSPID if they had at time of initial diagnosis an indeterminate diagnostic outcome: [1] sweat chloride < 30 mmol/L and 2 CFTR mutations with 0–1 CF-causing CFTR mutations; or [2] sweat chloride 30–59 mmol/L and < 2 CF-causing CFTR mutations [1, 2]. As a comparator group, NBS-positive infants with a clear-cut diagnosis of CF were also enrolled during the same period. Infants who presented with meconium ileus and fulfilled the diagnostic criteria for CF but were negative on NBS (i.e. false negative) were also included as part of the comparator group. Immunoreactive trypsinogen levels obtained at NBS from NBS-positive infants with CRMS/CFSPID and the comparator group of infants with CF were collected for the study.

In our clinical-research protocol, subjects with CRMS/CFSPID were clinically monitored and sweat tested 6 monthly in the first 2 years of life, and annually thereafter. Genotype information was obtained from NBS mutation analyses. Subjects would undergo confirmatory or further genotyping including CFTR gene sequencing, especially for infants identified with less than two CFTR mutations, usually prior to or at time of initial visit/diagnosis. Some of the CRMS/CFSPID subjects were subsequently reclassified as CF during the follow-up period (CRMS/CFSPID→CF) if they at a later time point fulfilled the diagnostic criteria for CF (either repeat sweat chloride levels ≥60 mmol/L or abnormal genotype from reassignment of previously indeterminate CFTR mutation to “CF-causing”) i.e. the participant’s diagnostic label and categorisation/re-categorisation in this prospective study occurred months after the initial diagnosis of CRMS/CFSPID and reflected real life reassignment of diagnosis in clinical practice.

### Statistical analysis

Descriptive statistics were presented according to the normality of the data distribution. Continuous data was presented as mean with standard deviation (SD) or median (interquartile range). Comparisons of NBS IRT concentrations were made using the Mann-Whitney test. IBM SPSS Statistics (v 23.0; SPSS Inc., Chicago, Illinois) were used. *P*-values < 0.05 were considered statistically significant.

## Results

### Subject characteristics

Out of 218 subjects, 98 CRMS/CFSPID and 120 CF subjects were enrolled. Among the 98 CRMS/CFSPID subjects, 97 subjects had two mutations (no more than one is CF-causing) with intermediate (*n* = 47) or normal (*n* = 50) sweat chloride, and 1 subject had one mutation (CF-causing) with intermediate sweat chloride. Mutations were either identified by NBS and/or by confirmatory or further genotyping via the CF clinic prior to or at time of initial visit/diagnosis. During the study period, 14 (14.3%) CRMS/CFSPID subjects fulfilled the diagnostic criteria for CF (CRMS/CFSPID→CF) (Table 1 and Fig. 1), while the diagnosis remained uncertain (CRMS/CFSPID→ CRMS/CFSPID) in 84 (85.7%) subjects. Among the 14 subjects with CRMS/CFSPID→CF, 10 had subsequent elevations of sweat chloride levels ≥60 mmol/L (with 5 based on abnormal sweat test alone and 5 based on both abnormal sweat test and genotype), and 4 based on abnormal genotype alone (Table 1 and Fig. 1). Among the 10 (out of 14) CRMS/CFSPID→CF patients who had abnormal sweat test levels (≥60 mmol/L) on follow-up sweat testing, sweat chloride increased to abnormal levels (mean [SD] of 68.30 [5.60] mmol/L) at a mean (SD) age of 35.97 (21.43) months.

**Table 1:**
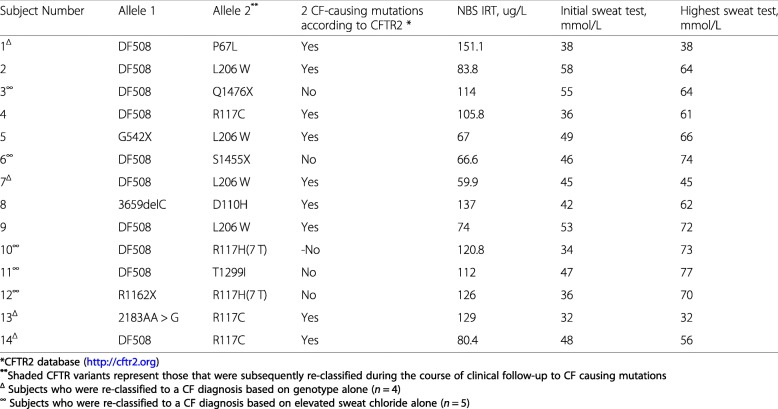
Characteristics of the 14 subjects with CFSPID who later fulfilled the diagnostic criteria for CF

### Immunoreactive trypsinogen levels

#### CRMS/CFSPID vs. CF

**Fig. 2:**
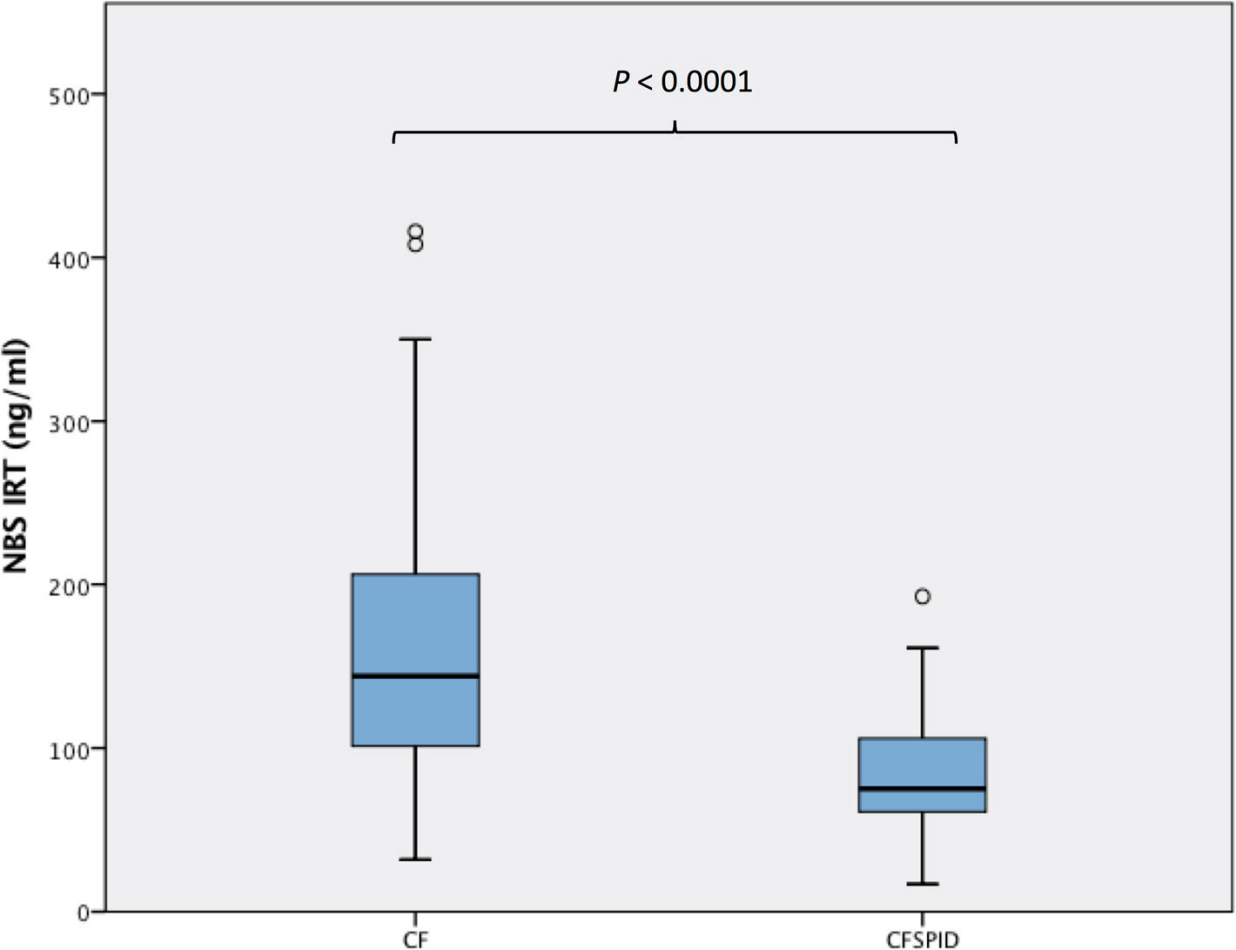
Comparison of newborn screening IRT levels between children with CF and CRMS/CFSPID

Infants with CF had significantly higher NBS IRT concentrations (ng/ml) (median (interquartile range)) than CRMS/CFPSID (143.8 (99.8–206.2) vs. 75.0 (61.0–105.9); *P* < 0.0001) (Fig. 2).

#### CRMS/CFSPID→CF vs. CRMS/CFSPID→CRMS/CFSPID

**Fig. 3:**
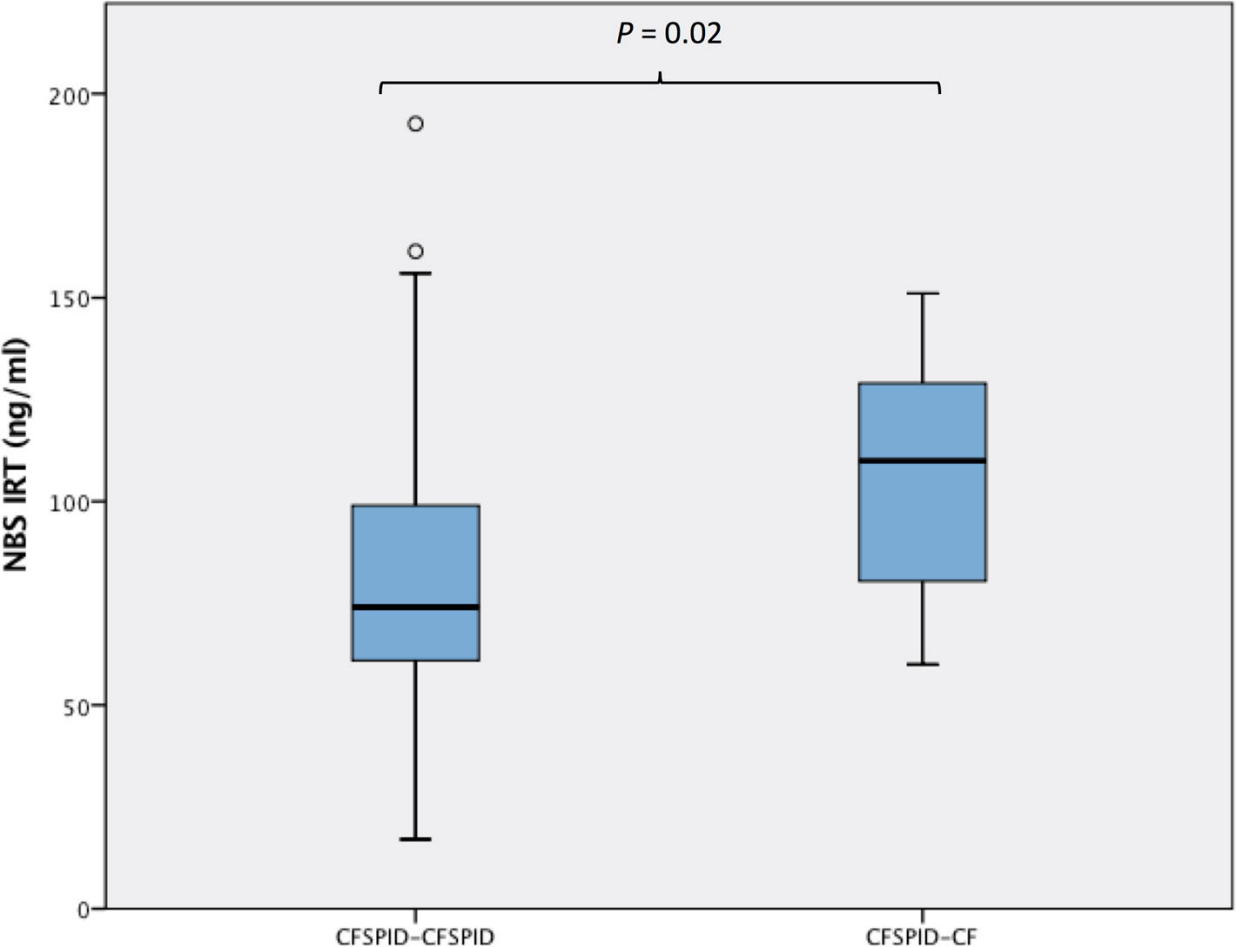
Comparison of newborn screening IRT levels between children with CRMS/CFSPID who subsequently met the criteria for CF (CFSPID→CF) and those whose diagnosis remained uncertain (CFSPID→CFSPID)

Infants with CRMS/CFSPID→CF (*n* = 14) had significantly higher NBS IRT concentrations (ng/ml) than CRMS/CFSPID→ CRMS/CFSPID (*n* = 83) (median (interquartile range): 108.9 (72.3–126.8) vs. 73.7(60.0–96.0); *P* = 0.02) (Fig. 3).

**Fig. 4:**
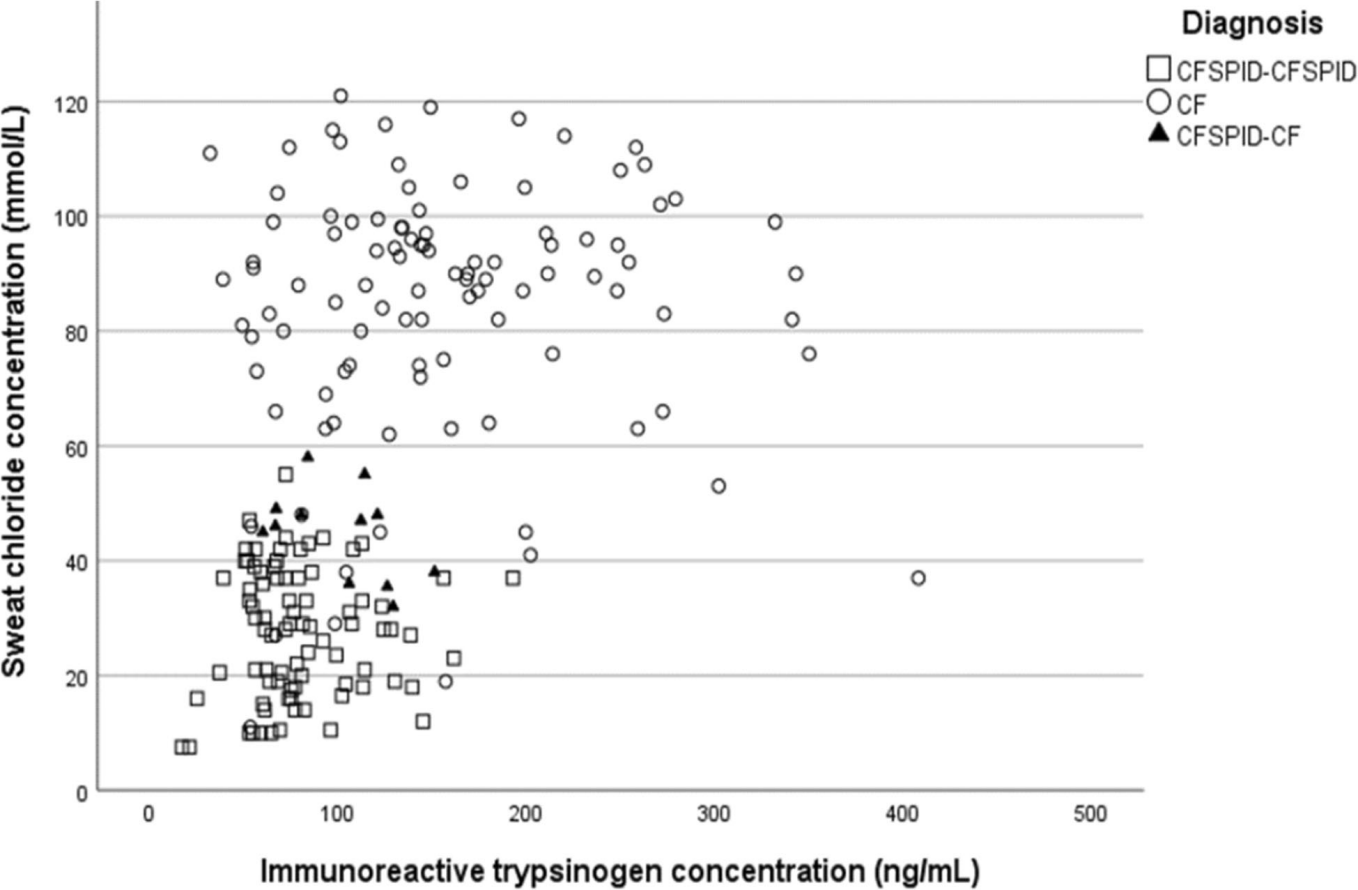
Initial sweat chloride measurements plotted against IRT levels for children with CF (represented by open circle = ○), children with CRMS/CFSPID who subsequently met the criteria for CF (CFSPID→CF; solid triangle = ▲) and those whose diagnosis remained uncertain (CFSPID→CFSPID; open square = □)

Further comparisons of the diagnostic outcomes are shown in Fig. 4 based on initial sweat chloride levels (i.e. done closest to NBS IRT) plotted against NBS IRT concentrations. While still overlapping with the CRMS/CFSPID→ CRMS/CFSPID cohort, infants with CRMS/CFSPID→CF aggregate much closer to the CF cohort compared to the overall CRMS/CFSPID→ CRMS/CFSPID group.

## Discussion

The outcomes of infants with CRMS/CFSPID remain uncertain. In this prospective longitudinal study of children with CRMS/CFSPID, we identified the potential role for NBS IRT as a biomarker to predict the likelihood of having CF, with significantly different IRT levels between CF and CRMS/CFSPID, and also between CRMS/CFSPID→CF and CRMS/CFSPID→ CRMS/CFSPID.

Our findings have biological plausibility. Among all organ systems affected by CF disease, the exocrine pancreas serves as a phenotypic barometer of CFTR function and correlates well with the underlying genotype [11–13]. The pancreatic insufficiency prevalence (PIP) scores, based on the exocrine pancreatic phenotype, have been shown to accurately distinguish the severity of different CFTR mutations [13]. In this study, we speculate that IRT levels reflect the severity of pancreatic disease and thus the severity of the underlying CFTR gene mutation, with higher levels during the first few years of life indicating more extensive pancreatic damage correlating with more severe CFTR dysfunction, akin to higher IRT levels in newborns with CF compared to non-CFs. Our observations are in keeping with the association between trypsinogen levels and the likelihood of having CF disease. However, this relationship is likely complex and non-linear (as observed in Fig. 3). Furthermore, in two separate studies, higher IRT levels were observed in children with two CF-causing mutations compared to those with either one mutation of varying clinical consequence [7] or one non-CF causing mutation [8]. These findings may be highly relevant clinically, as to date, aside from diagnostic tests such as repeat sweat testing, extensive genotyping or other functional CFTR testing, there is no test that predicts or stratifies the risk of a later CF diagnosis following an initial diagnosis of CRMS/CFSPID. In our previous study comparing CRMS/CFSPID→ CRMS/CFSPID and CRMS/CFSPID→CF groups, there were no significant differences in respiratory symptoms and microbiology present [10]. Furthermore, IRT levels are available at the time of diagnosis and thus limiting the delays for risk stratification.

The diagnosis of CF is a dynamic and evolving process among individuals with an indeterminate diagnosis. There is continuous new discovery on the pathogenicity of rare CFTR gene variants due to the ongoing efforts of the CFTR2 project (http://cftr2.org). Furthermore, we have previously demonstrated increases in sweat chloride concentrations over time in a subset of these individuals [10]. In this current study, all 14 subjects with an initial diagnosis of CRMS/CFSPID carried two CFTR variants. Of those, 9 infants were later reassigned a diagnosis of CF (CRMS/CFSPID→CF) based on genotype; 5 of these 9 subjects also had increases in sweat chloride levels above the 60 mmol/L cut-off for CF diagnosis with time while 4 of 9 subjects met the diagnosis of CF by genotyping alone. Thus our experience provides support for comprehensive genotyping in these individuals. While many of these individuals already had their 2nd CFTR mutation identified at time of assessment, these mutations were not considered disease-causing until later in the course of follow-up (with the roll-out of new pathogenic CFTR variants by CFTR2). Since CFTR2 is based on CF patient registries, it is worth noting though that the primary goal of CFTR2 is to identify the most common CF-causing variants rather than the most common non CF-causing variants, which is important in helping distinguish CRMS/CFSPID→ CRMS/CFSPID from CRMS/CFSPID→CF in the long-term. The remaining 5 subjects later fulfilled the diagnosis of CF based on sweat testing alone. As this was a prospective, longitudinal study all these 14 individuals were classified as CRMS/CFSPID and not CF at the time of presentation and recruitment (i.e. as it occurred in real-life clinical practice).

The present study has limitations. Our observations are based on a small sample size, particularly with the CRMS/CFSPID→CF cohort despite the multicentre and multiyear approach of this study. Future efforts would include confirming our observation in another cohort and then either defining a sensitive IRT cut-off or identifying trajectory of longitudinal IRT levels (i.e. levels over time or age) to predict risk of developing CF based on a larger sample size. Due to the multicentre and multinational approach, subjects were identified based on different NBS protocols used. This study also did not account for variability in IRT levels within and across different provinces, and variations in IRT concentration among different races, birth weight and gestational age. However, the phenomenon of identifying CRMS/CFSPID is ubiquitous irrespective of the variations in NBS algorithms and despite this, there were significant differences in IRT levels between the different cohorts of children.

In conclusion, predicting outcomes of children with CRMS/CFSPID remains a challenge, requiring careful monitoring over time. Newborn screening IRT levels are significantly different between children with CRMS/CFSPID who subsequently met the criteria for CF and those whose diagnosis remained uncertain, and may have the potential to predict the likelihood of later fulfilling the diagnostic criteria for CF.

## Data Availability

The datasets generated and/or analysed during the current study are not publicly available as the study is ongoing.

## Abbreviations

CF: Cystic fibrosis
CFSPID: Cystic fibrosis screen positive inconclusive diagnosis
CFTR: CF transmembrane conductance regulator
CRMS: CFTR-related metabolic syndrome
IRT: Immunoreactive trypsinogen
NBS: Newborn screening
